# Neonicotinoid insecticides can serve as inadvertent insect contraceptives

**DOI:** 10.1098/rspb.2016.0506

**Published:** 2016-07-27

**Authors:** Lars Straub, Laura Villamar-Bouza, Selina Bruckner, Panuwan Chantawannakul, Laurent Gauthier, Kitiphong Khongphinitbunjong, Gina Retschnig, Aline Troxler, Beatriz Vidondo, Peter Neumann, Geoffrey R. Williams

**Affiliations:** 1 Institute of Bee Health, Vetsuisse Faculty, University of Bern, Bern, Switzerland; 2 Veterinary Public Health Institute, Vetsuisse Faculty, University of Bern, Bern, Switzerland; 3 Environmental Science Department, University of Koblenz-Landau, Landau, Germany; 4 Bee Protection Laboratory (BeeP), Department of Biology, Faculty of Science, Chiang Mai University, Chiang Mai, Thailand; 5 Agroscope, Swiss Bee Research Centre, Bern, Switzerland; 6 School of Science, Mae Fah Luang University, Chiang Rai, Thailand; 7 Department of Zoology and Entomology, University of Pretoria, Pretoria, South Africa

**Keywords:** *Apis mellifera*, insecticide, pollination, reproduction, sperm, sub-lethal

## Abstract

There is clear evidence for sublethal effects of neonicotinoid insecticides on non-target ecosystem service-providing insects. However, their possible impact on male insect reproduction is currently unknown, despite the key role of sex. Here, we show that two neonicotinoids (4.5 ppb thiamethoxam and 1.5 ppb clothianidin) significantly reduce the reproductive capacity of male honeybees (drones), *Apis mellifera*. Drones were obtained from colonies exposed to the neonicotinoid insecticides or controls, and subsequently maintained in laboratory cages until they reached sexual maturity. While no significant effects were observed for male teneral (newly emerged adult) body mass and sperm quantity, the data clearly showed reduced drone lifespan, as well as reduced sperm viability (percentage living versus dead) and living sperm quantity by 39%. Our results demonstrate for the first time that neonicotinoid insecticides can negatively affect male insect reproductive capacity, and provide a possible mechanistic explanation for managed honeybee queen failure and wild insect pollinator decline. The widespread prophylactic use of neonicotinoids may have previously overlooked inadvertent contraceptive effects on non-target insects, thereby limiting conservation efforts.

## 1. Introduction

Factors affecting reproductive success have a profound influence not only on a single individual's fitness, but on the dynamics of entire populations [1,2]. This principle provides a framework for pest control strategies that target reproduction. For example, modern-day agricultural practices frequently demand intensive insect pest management to ensure high-quality crops [3,4]. Strategies such as sterile insect techniques and insect growth regulator insecticides are designed for their sublethal effects on adult insect reproduction [5–7], whereas others may kill the pest insect outright [8,9].

Advances in agrochemical research highlight a lack of knowledge of the sublethal effects of insecticides on their target insect pests [10], as well as on sympatric beneficial insects such as bees that provide vital ecosystem services [11–13]. Frequently applied neonicotinoid insecticides can affect the nervous system of insects by acting as agonists of postsynaptic nicotinic acetylcholine receptors [14–16]. Recently, they have been shown to elicit sublethal effects on several bee genera, such as impairing bumblebee queen (primary reproductive females) production and diminishing honeybee queen reproduction [17,18]. However, to date no data exist on how neonicotinoid insecticides may affect male insect reproduction.

Historically, the honeybee (*Apis mellifera*) has served as a model insect to investigate the effects of various anthropogenic and environmental stressors [9] because it can be easily maintained and is relatively well studied. Furthermore, honeybees contribute essential pollination services to agriculture [19] and wild plants [20]. Queens perform mating flights soon after emergence to collect and store sufficient quantities of sperm from multiple drones (male sexuals) to last their lifetime [21]. This highly polyandrous strategy [22] conveys several benefits, including increased colony functioning and resistance to disease [23–25].

Within the last decade, honeybees have experienced severe annual mortalities in the Northern Hemisphere [26], probably because of a diverse array of stressors acting in concert [20,27]. These events have paralleled declines of wild bees [28,29]. It is believed that poor queen health (i.e. premature queen replacement, frequent unfertilized egg-laying) is a major contributor to honeybee colony mortality [30,31], yet factors affecting honeybee reproductive success remain largely unexplored. Recent studies have demonstrated, however, that miticides can affect the production and storage of honeybee sperm in males [32–34] and stored sperm by mated females [35], respectively. Because queen survival and productivity are intimately connected to successful mating, any influence on sperm quality may have profound consequences for the fitness of the queen, as well as the entire colony [36–39].

Here, we tested for the first time the effects of neonicotinoid insecticides on male insect reproduction. We employed honeybee drones as models that were exposed during development to chronic field-realistic concentrations of the neonicotinoids thiamethoxam and clothianidin. We hypothesized that drones reared in colonies exposed to neonicotinoids would experience significant lethal (reduced longevity) and sub-lethal (sperm quality) effects compared with drones from control colonies based on previous studies demonstrating strong sublethal effects of neonicotinoids on female insect reproduction [17,18,30,40] and longevity [41–43], and because insecticide-induced reactive oxidative stress has been shown to reduce sperm quality [44–47].

## 2. Material and methods

The study was performed in Bern, Switzerland, between April and September 2015 using 20 *A. mellifera* L. honeybee colonies that were established at the beginning of the experimental period using the shook swarm method [48] to source drones and workers (primarily non-reproductive females). Each colony initially consisted of one laying sister queen, 1.8 kg workers, as well as five Dadant frames (each 435 mm by 298 mm) containing organic worker cell wax foundation that was tested for a broad array of agricultural chemical residues by the University of Hohenheim; an additional frame containing organic drone cell wax foundation was added approximately three weeks later to promote drone production [49].

### (a) Insecticide exposure

In early May 2015, colonies were randomly assigned to one of two treatments (insecticide or control). Each colony was provided daily with 100 g pollen paste (60% fresh honeybee corbicular pollen, 10% organic honey, and 30% powder sugar) according to Sandrock *et al.* [50] and Williams *et al.* [18]. Pollen paste for insecticide colonies additionally contained 4.5 ppb thiamethoxam and 1.5 ppb clothianidin (both Sigma-Aldrich), which represents field-realistic concentrations found in plant pollen [51]; applied concentrations were confirmed (4.9 ppb thiamethoxam and 2.1 ppb clothianidin in insecticide patties; below the limit of quantification for thiamethoxam (less than 0.02 ppb) and clothianidin (less than 0.08 ppb) in control patties) by the French National Centre for Scientific Research using ultra-high performance liquid chromatography-tandem mass spectrometry (UHPLC-MS/MS). Pollen paste feeding occurred over a period of 50 days to ensure colonies would be exposed to at least two complete brood cycles. Recent evidence suggests that foraging honeybees may be exposed to insecticide residues for a similar period due to contamination of non-agricultural foraging areas by surface run-off or drainage from nearby treated crops [52,53]. During the entire period, each colony was equipped with an entrance pollen trap to partially restrict forager-collected corbicular pollen entering the hive in order to promote pollen paste feeding [50].

### (b) Source of drones and workers

Thirty-eight days post-initial pollen paste feeding, queens of each colony were first caged for approximately 48 h to a drone brood frame, and then 1 day later to a worker brood frame for an additional approximately 48 h to obtain sufficient numbers of drones and workers of the same known age cohort. Both experimental brood frames remained within their corresponding colonies until approximately 24 h prior to simultaneous drone and worker emergence; frames were then transferred to a laboratory incubator maintained in complete darkness at 34.5°C and 60% relative humidity [54].

### (c) Teneral body mass and cage mortality

Upon emergence, each experimental drone and worker was visually examined to assess for physical abnormalities and the presence of the parasitic mite *Varroa destructor*. For each colony, the first 30 drones to emerge, which were free of *V. destructor* infestation and abnormalities, were weighed to the nearest 0.1 mg using an analytic scale (Mettler Toledo AT400). These drones, plus the next 30 of similar status (no *V. destructor* or abnormalities) to emerge per colony, were then placed in standard hoarding cages (250 cm^3^) [54] corresponding to their source colony (and, therefore, respective treatment groups, i.e. insecticide or control). In total, each colony provided six hoarding cages of bees that each contained 10 drones and 20 workers from the same colony. The presence of workers in each cage was necessary because drones depend on worker attendance within the first few days of emergence [55–57]. Cages were subsequently maintained in complete darkness at 30°C and 60% relative humidity [54], and given 50% (w/v) sucrose solution and pollen paste (60% fresh honeybee corbicular pollen and 40% sugar powder) *ad libitum* to provide a carbohydrate energy source and ample proteins for organ and tissue development [58,59], respectively. Food was replaced every 72 h, whereas cage mortality was recorded every 24 h; dead individuals were removed using a forceps. After 8 days, all cages were exposed to indirect natural light for 1 h to promote and imitate an initial orientation flight [21]. The assay was terminated immediately after all experimental drones died.

### (d) Sperm assessment

Three cages per colony were randomly selected to assess drone sperm quantity and viability at 14 days post-cage assay initiation, the typical age drones reach sexual maturity [60,61]. Drones in these cages were carefully removed using a forceps; to prevent sperm from migrating into the penis bulb, the drones were dissected alive by pinning them onto a wax plate [62]. Following Carreck *et al.* [63] the testes, mucus glands, and seminal vesicles were removed from each drone, placed in a 1.5 ml Eppendorf^®^ tube containing 500 µl Kiev^+^ buffer, and crushed to form a diluted stock sperm solution.

Immediately, a 50 µl aliquot of the stock sperm solution was set aside in a separate 1.5 ml Eppendorf^®^ tube for analyses of sperm viability (proportion of sperm alive [64]). Sperm viability was quantified using the method previously described by Collins and Donoghue [65] and Stürup *et al.* [66]. In brief, each sample was diluted with 50 µl of Kiev^+^ buffer before 2 µl of propidium iodide (PI) solution (1 mg ml^−1^) and 1 µl of Hoechst 33342 (0.5 mg ml^−1^) [67] (both Sigma-Aldrich) were added to the suspension. Samples were then incubated for approximately 20 min in complete darkness and then gently vortexed. Ten microlitres were viewed at 400× magnification using a fluorescent microscope (Olympus BX41, Switzerland) equipped with filter cubes for UV excitation [67]. Ten visual fields were selected for each sample so that the quantity of living and dead sperm could be counted; an average value was then calculated from these fields [67].

In addition, 20 µl of each stock sperm solution were diluted with 80 µl Kiev^+^ buffer (1 : 5 dilution) in a 1.5 ml Eppendorf^®^ tube to perform sperm counts. Sperm densities were measured using a Neubauer counting chamber under light microscopy (Thermo Fischer Scientific, USA). The final density of sperm was quantified using the following calculation [68]: total sperm quantity (500 µl) = average number of sperm counted in two Neubauer counting chambers × dilution factor (1 : 5) × sperm volume used for Neubauer counting chamber (10 µl) × stock solution volume (500 µl). Once both total sperm quantity and sperm viability were assessed, the total living sperm quantity was obtained by multiplying the two together.

### (e) Statistical analyses

Three-level generalized regression mixed models with random intercepts were fitted using STATA14 [69], wherein individual drones were considered independent units, treatment (insecticide versus control) was included as the fixed term (or explanatory variable) and colonies and cages as random effects because of the clustering of individuals [70]. All statistical figures were created using NCSS v. 9.0.15 [71].

Drone teneral body mass was normally distributed (Shapiro–Wilk's test for normality, *p* = 0.44), so a general linear model was fitted using the meglm function. Total sperm quantity and the total living sperm quantity are count data, and were not normally distributed (Shapiro–Wilk's test for normality, *p* < 0.001) so were therefore fitted to negative binomial models using the menbreg function. Sperm viability is a score ranging from 0 to 100% and was also not normally distributed (Shapiro–Wilk's test for normality, *p* < 0.001) so an ordered logistic model was employed [72]. We used an XY scatter plot and Spearman's correlation coefficient to assess a possible correlation between sperm quantity and sperm viability. Lastly, survival times of drones and workers for both treatments were fitted using the mestreg function for multilevel survival models [70]. Median longevity was calculated as the 50th percentile of the survival time [73]. Drones sampled on day 14 for sperm assessments, as well as their accompanying caged workers, were censored. Whenever possible, every three-level model was compared with its single-level model counterpart using a likelihood ratio (LR) test [69]. LR tests, which do not rely on the assumption of asymptotic normal sampling distributions, can be used to demonstrate which model best fit the data.

Median differences and their 95% CI were calculated using the STATA14 package somersd. The function cendif calculates confidence intervals for Hodges–Lehmann median differences (or other percentile differences) between two groups [74].

## 3. Results

### (a) Teneral body mass and cage mortality

**Figure 1.**
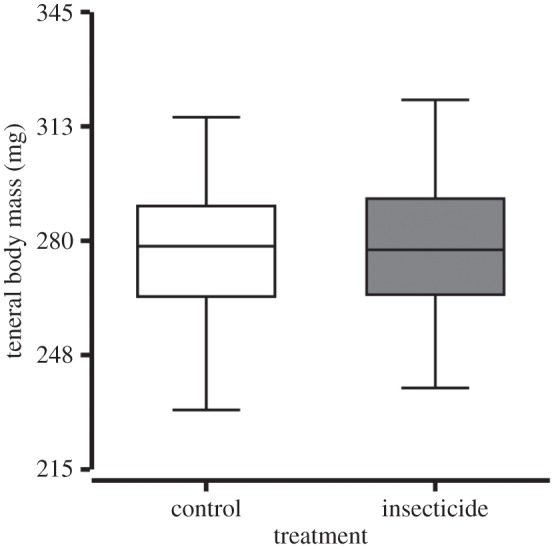
Drone (male) honeybee teneral body mass. Comparison of drone honeybee (*Apis mellifera*) teneral body mass (mg) showed no significant difference between controls (*N* = 200) and neonicotinoid insecticides (*N* = 120) (*p* = 0.80). The boxplots show the inter-quartile range (box), the median (black line within box), data range (horizontal black lines from box), and outliers (black dots).

**Figure 2.**
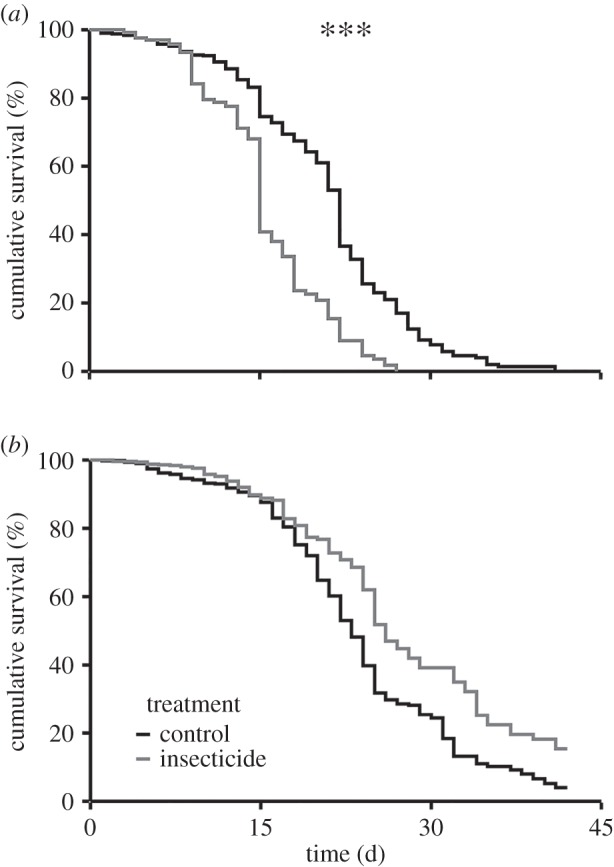
Honeybee drone (male) and worker (female) cage mortality. Survival curves (Kaplan–Meier) indicate the cumulative survival (%) of honeybee (*Apis mellifera*) drones (*N* = 567) (*a*) and workers (*N* = 1120) (*b*) under neonicotinoid insecticide exposure compared with controls. A significant difference was only observed for the mortality of drones (*p* < 0.001). A significant difference between treatment groups is indicated by ***p < 0.001.

No significant difference was observed between treatments for drone teneral body mass (*p* = 0.80; figure 1), which was 277.06 ± 17.06 mg and 278.27 ± 18.16 mg for the controls and insecticides, respectively (mean ± standard error (s.e.)). However, median longevity of insecticide drones (15 ± 15–15 days) was significantly lower than controls (22 ± 21–22 days) (*p* < 0.001; median ± 95% CI; figure 2*a*). Furthermore, insecticide drone survival was significantly reduced compared with controls for up to 14 days (point of sexual maturity); mortality was 16.82 ± 0.02% and 32.08 ± 0.03% for controls and insecticides, respectively, which represents an approximately 50% difference (*p* < 0.001; cumulative hazard% ± s.e.; figure 2*a*). By contrast, no significant difference in worker median longevity was observed between controls (23 ± 22–24 days) and insecticides (26 ± 25–29 days) (*p* = 0.27; median ± 95% CI; figure 2*b*).

### (b) Sperm assessment

**Figure 3.**
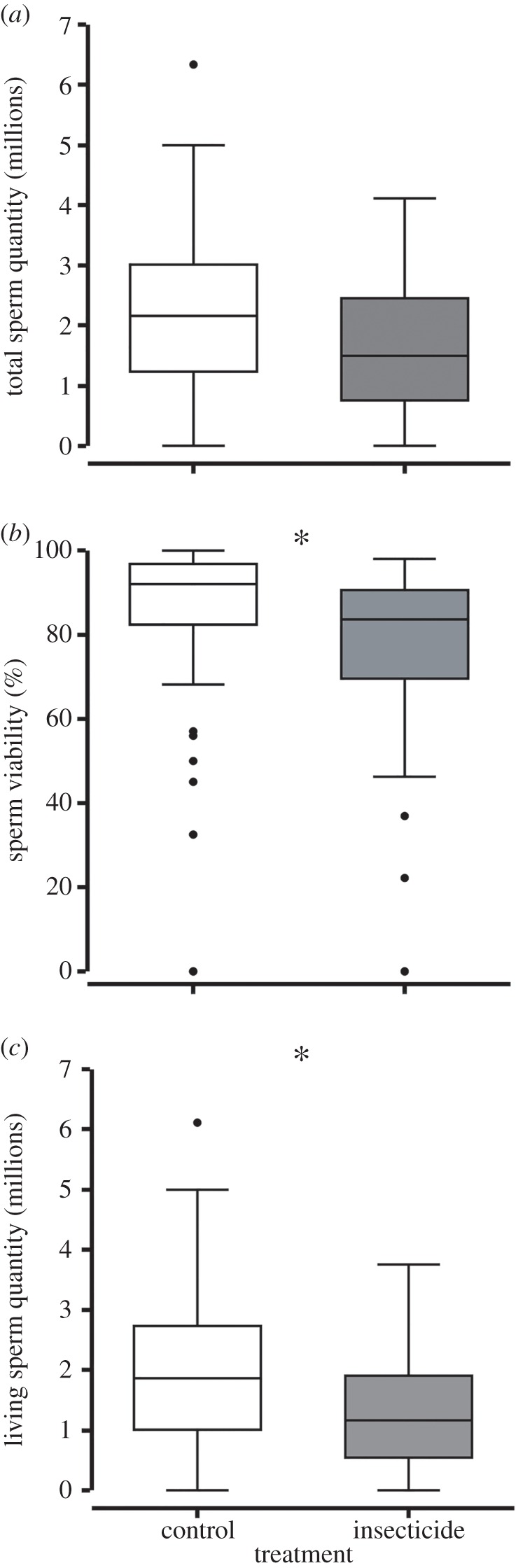
Honeybee sperm assessment. Assessment of various sperm traits in male (drone) honeybees (*Apis mellifera*) under neonicotinoid insecticide (*N* = 90) exposure compared with controls (*N* = 145). (*a*) Comparison of sperm quantity showed no significant differences (*p* = 0.1375). (*b*) Percentage of viable sperm in honeybee drones showed significant differences (*p* = 0.03). (*c*) Total quantity of living sperm in honeybee drones showed a significant difference (*p* < 0.05). All boxplots show the inter-quartile range (box), the median (black line within box), data range (horizontal black lines from box), and outliers (black dots). A significant difference between treatment groups is indicated by *p < 0.05.

No evidence of treatment effect was found between control (2.19 ± 1.93–2.55 million) and insecticide (1.55 ± 1.33–2.05 million) drone sperm quantity 14 days post-cage assay initiation (*p* = 0.14; median ± 95% CI; figure 3*a*). By contrast, sperm viability was significantly different between the two treatment groups, with insecticide drones having 8 ± 4.6–11.3% (median difference ± 95% CI) lower sperm viability than controls (*p* = 0.03; figure 3*b*). Sperm viability was 92 ± 90–94% and 83.5 ± 80–86% in the controls and insecticides, respectively (median ± 95% CI). No correlation was observed between sperm quantity and sperm viability (Spearman's *|r|* = 0.05, *p* = 0.44). In addition, a significant difference was observed between control (1.98 ± 1.72–2.18 million) and insecticide (1.2 ± 0.20–1.6 million) treatments for total living sperm quantity (*p* < 0.05; median ± 95% CI; figure 3*c*), which represents on average approximately 39% less living sperm in insecticides compared with controls. The median difference and its 95% CI was 0.61 ± 0.32–0.90 million less living sperm in insecticides compared with controls.

## 4. Discussion

Factors governing reproductive success have a profound influence on shaping populations by affecting fitness [1,75]. Bountiful examples in nature include predation and parasitism [76,77]; however, anthropogenic influences such as industrial pollution and landscape fragmentation may also be important drivers [78–80]. Neonicotinoid insecticides represent a class of neurotoxins widely employed in agriculture for insect pest control [15]. Our study clearly demonstrates that neonicotinoid insecticides can have significant lethal (lifespan) and sublethal (sperm viability and living sperm quantity) effects on honeybee drones. Using the honeybee as a model, we hereby provide the first evidence that field-relevant concentrations of these chemicals can elicit effects on male insect reproductive capacity.

For eusocial insects such as honeybees, polyandry conveys several fitness benefits, such as reducing parasitism [81,82], buffering colony performance against environmental change [83], and improving task efficiency [84–86]; it also ensures sufficient sperm to maintain long-living queens and large colonies [85,87]. Therefore, evidence to suggest that neonicotinoids can impair reproduction provides one possible explanation for recent observations of increased annual mortality of managed honeybees [17,29,30,88], as well as the general decline of wild insect pollinators [29,89], throughout the Northern Hemisphere. Although drones (male honeybees) do not directly contribute to colony survival [90], their role via mating is vital for colony fitness [91]. Furthermore, queen survival and productivity are intimately connected to proper mating as the depletion of sperm results in costly replacement of the queen by the colony, which can only successfully occur during specific periods of the year [92]. Recent data linking poor queen health to colony mortality [30], possibly because of low quality stored sperm from stressors such as miticides or insect growth regulator insecticides [33,93,94], highlight the urgent need for investigating possible factors that may affect male reproductive success among non-target, beneficial insects.

Honeybee teneral body mass immediately succeeding pupation is often used as an index for an individual's overall condition [95,96]; both pathogens and insecticides reduce teneral body mass [43,97,98]. Our data revealed the teneral body mass of drones was not influenced by neonicotinoids, despite a previous investigation demonstrating reduced mass of neonicotinoid-exposed teneral workers [43]. Reasons for this disparity could be due to differences in neonicotinoid chemistries (the neonicotinoids, thiamethoxam and clothianidin versus imidacloprid), and routes of exposure (pollen versus sugar water). Nonetheless, our results demonstrated that neonicotinoid exposure strongly reduces the longevity of drones. Considering that sexual maturity is typically reached 9–14 days post-emergence, approximately 30% of neonicotinoid-exposed drones in our study would likely not be afforded the opportunity to mate with virgin queens. This could have severe consequences for colony fitness [99,100], as well as reduce the overall genetic variation within honeybee populations [101]. Conversely, female workers exposed to neonicotinoids did not experience a reduction in longevity, despite contrary evidence from previous studies [42,102]. This again could be due to differences among experimental treatments (the neonicotinoids, thiamethoxam and clothianidin versus thiacloprid), cage assay conditions (e.g. sugar and pollen feeding versus only sugar), or treatment exposure (colony versus individual level). This may, furthermore, be explained by the haploid–diploid susceptibility hypothesis, which proposes that hemizygous haploid individuals such as honeybee drones may experience increased susceptibility to environmental stressors due to decreased genetic variability [98,103]. Recent studies revealed that agrochemicals are capable of impairing immune function [104–107]; therefore, it is possible that neonicotinoid-exposed drones possess reduced detoxification abilities that subsequently affected lifespan.

The successful transfer of male sperm is the primary goal of copulation [23]. Therefore, honeybee mating success is highly dependent upon drones producing large quantities of sperm that must remain in excellent condition for an extended period within the queen's sperm storage organ (spermatheca). Although storage conditions afforded by the queen are important to ensuring long-term sperm survival [47], sperm received from the drone must nonetheless be of high quality. Even though neonicotinoids did not appear to influence the quantity of total sperm produced by males, we did observe a significant negative effect on sperm viability, which in turn resulted in a significant reduction in the number of living sperm produced by neonicotinoid drones. It is possible that this observation could be caused by reactive oxidative stress affecting sperm [44,46,47]; this possible mechanism should be studied in the future. The mean sperm quantity observed in this study was lower than found in previous cage and field studies [36,61,108,109]. The lower values could have resulted from laboratory cage conditions [36], as well as conditions of the drones during development [110].

Although only a small proportion of transferred sperm is stored by the queen [111], any decrease in sperm quality could have negative consequences [112]. Aided by muscular contractions in the female reproductive tract, transferred sperm actively swim from the oviducts to the female spermatheca, a process that can take up to approximately 40 h [60,111]. Considering that the majority of queen mating flights occur within 2–4 days [21,22,113], poor-quality sperm received during mating could result in a reduced quantity of stored sperm, or in extended, risky mating flight periods to ensure sufficient sperm is obtained [50,60,114,115].

As the primary egg layer and an important source of colony cohesion, the queen is intimately connected to colony performance [30]. Increased reports of queen failure have recently been reported in North America and Europe [30,31,116]; however, no studies have so far investigated the role of neonicotinoids and male health to explain this phenomenon. For the first time, we have demonstrated that frequently employed neonicotinoid insecticides in agro-ecosystems can elicit important lethal (reduced longevity) and sublethal (reduced sperm viability and living sperm quantity) effects on non-target, beneficial male insects; this may have broad population-level implications [17,29,117]. Furthermore, the observed effects of neonicotinoid insecticides on a highly polyandrous bee species are particularly worrying for monandrous insects that rely on a single successful mating event to provide fertilized eggs [118].

By demonstrating the effects of neonicotinoid insecticides on male insect reproduction, our study provides a possible mechanism, in addition to introduced parasites and other land-use practices, for honeybee queen failure [30,31] and a general decline of non-target beneficial insects throughout the Northern Hemisphere. Considering that neonicotinoid insecticides can affect non-target male vertebrate reproduction [119–122], our complementary findings for invertebrates are not surprising. Our research further highlights the urgent need for thorough investigations of possible unintended effects of agricultural insecticides on male insect reproductive traits, particularly among sympatric beneficial non-targets. For instance, it is not known if the insecticides had a direct effect on the male's reproductive traits via contaminated pollen, or an indirect effect because of poor nursing quality and reduced hypopharyngeal gland activity of young, exposed workers [123,124]. Furthermore, future research should be directed towards understanding how our results relate to broader implications for honeybee reproduction in the natural environment. Although recent improvements to regulatory requirements for evaluating the environmental impacts of insecticides have been adopted, none so far directly address the reproduction of beneficial insects [9].

## Supplementary Material

Table Summary of Results

## Data accessibility

The complete raw data can be found at the Dryad repository. See doi:10.5061/dryad.bs515.
