# Supplementary material for: Neonicotinoid insecticides can serve as inadvertent insect contraceptives
Source: Proc Biol Sci. 2016 Jul 27;283(1835):20160506. doi: 10.1098/rspb.2016.0506 (PMC4971197; doi:10.1098/rspb.2016.0506)
Supplement: Table Summary of Results [file rspb20160506supp1.docx]

|  |  |  |  |  |  | **Quantiles** | | | | |
| --- | --- | --- | --- | --- | --- | --- | --- | --- | --- | --- |
| **Caste** | **Variable** | **Treatment** | **Sample Size** | **Mean** | **S.D.** | **Min.** | **0.25** | **Median** | **0.75** | **Max.** |
| **Drones** |  |  |  |  |  |  |  |  |  |  |
|  | Teneral Body Mass (mg) | Control | 200 | 277.06 | 17.06 | 232 | 264.2 | 278.35 | 290 | 315 |
|  |  | Pesticide | 120 | 278.27 | 18.16 | 238.1 | 264.75 | 277.4 | 292 | 320 |
|  | Drone Survival (d) | Control | 327 | 16.79 | 0.31 | 1 | 15 | 22 | 25 | 41 |
|  |  | Pesticide | 240 | 14.05 | 0.36 | 3 | 13 | 15 | 18 | 41 |
|  | Sperm Quantity | Control | 145 | 2100000 | 1300000 | 0 | 1200000 | 2200000 | 3000000 | 6300000 |
|  |  | Pesticide | 90 | 1300000 | 920000 | 0 | 550000 | 1200000 | 1900000 | 3800000 |
|  | Sperm Viability (%) | Control | 139 | 87.05 | 15.98 | 0 | 82.42 | 91.91 | 96.81 | 100 |
|  |  | Pesticide | 86 | 78.23 | 19.12 | 0 | 69.75 | 83.59 | 90.53 | 98.03 |
|  | Living Sperm | Control | 139 | 2000000 | 1200000 | 0 | 1100000 | 200000 | 2700000 | 6100000 |
|  |  | Pesticide | 86 | 1400000 | 920000 | 0 | 590000 | 1200000 | 1900000 | 3800000 |
| **Workers** |  |  |  |  |  |  |  |  |  |  |
|  | Worker Survival (d) | Control | 640 | 17.96 | 0.26 | 1 | 19 | 23 | 30 | 41 |
|  |  | Pesticide | 480 | 18.06 | 0.3 | 1 | 21 | 26 | 35 | 41 |

**Table 1. Summary of Results.** Summary statistics of the different variables for insecticide drones when compared to the control group. Median lifespans and their quartiles are calculated by means of censured survival models.

**Table 2. Summary of statistical methods and results.** Summary of the STATA14 functions used to fit three-level models, the outcome variable, the type of regression coefficient estimated (IRR: incidence rate ratio, OR: odds ratio, HR: hazard ratio), the P-value, the estimated coefficient of pesticide with respect to control and its 95% CI.

|  |  |  | |  | |  | **Regression Coefficient (RC)** | | **95 % Confidence Interval** | |
| --- | --- | --- | --- | --- | --- | --- | --- | --- | --- | --- |
| **Caste** | **Variable** | | **Treatment** | | **STATA14 function** | **P-Value** | **Type** | **RC Value** | **Lower** | **Upper** |
| **Drones** |  | | | | | | | | | |
|  | Teneral Body Mass (mg) | | Control | | megln | 0.80 | Linear | 1.40 | -11.22 | 14.03 |
|  |  | | Pesticide | |  |  |  |  |  |  |
|  | Drone Survival (d) | | Control | | mestreg | 0.001 | HR | 2.17 | 1.13 | 4.15 |
|  |  | | Pesticide | |  |  |  |  |  |  |
|  | Drone Survival at day 14 (d) | | Control | | mestreg | 0.001 | HR | 2.36 | 1.28 | 4.35 |
|  |  | | Pesticide | |  |  |  |  |  |  |
|  | Sperm Quantity | | Control | | menbreg | 0.13 | IRR | 0.79 | 0.59 | 1.08 |
|  |  | | Pesticide | |  |  |  |  |  |  |
|  | Sperm Viability (%) | | Control | | meologit | 0.03 | OR | 0.29 | 0.09 | 0.87 |
|  |  | | Pesticide | |  |  |  |  |  |  |
|  | Quantity Living Sperm | | Control | | menbreg | 0.05 | IRR | 0.69 | 0.48 | 1.00 |
|  |  | | Pesticide | |  |  |  |  |  |  |
| **Workers** |  | | | | | | | | | |
|  | Worker Survival (d) | | Control | | mestreg | 0.27 | HR | 0.72 | 0.40 | 1.30 |
|  |  | | Pesticide | |  |  |  |  |  |  |
